# Impact of serum TRAb level changes on the efficacy of ^131^I therapy in Graves’ disease: a decision tree prediction model

**DOI:** 10.3389/fendo.2025.1581353

**Published:** 2025-07-01

**Authors:** Ziyu Ma, Nan Liu, Yiwang Du, Xuan Wang, Xue Li, Shasha Hou, Zhaowei Meng, Jian Tan, Wei Zheng

**Affiliations:** ^1^ Department of Nuclear Medicine, Tianjin Medical University General Hospital, Tianjin, China; ^2^ Department of Rheumatology and Immunology, Tianjin Medical University General Hospital, Tianjin, China

**Keywords:** Graves’ disease, radioactive iodine therapy, TRAb, decision tree, efficacy, prediction model

## Abstract

**Objectives:**

This study aims to evaluate the relationship between changes in thyroid stimulating hormone receptor antibody (TRAb) levels before and after treatment and the efficacy of radioactive iodine therapy (RAI) for Graves’ disease (GD). Additionally, a decision tree model was developed to predict treatment outcomes based on variations in serum TRAb levels.

**Methods:**

A total of 728 patients were evaluated to investigate the association between TRAb level fluctuations and RAI treatment efficacy. A decision tree model was constructed using TRAb level changes at 3 and 6 months post-RAI to predict clinical outcomes.

**Results:**

Among the 728 patients, 326 (44.8%) achieved clinical remission. Patients with lower TRAb levels at 18 months post-RAI exhibited higher remission rates, particularly those whose TRAb levels returned to baseline. A greater decline in TRAb levels between 18 and 36 months post-RAI was also correlated with improved treatment outcomes. Decision tree models based on TRAb level changes at the 3rd and the 6th month post-RAI demonstrated predictive accuracies of 74.36% and 72.46%, respectively. Further analysis showed that patients with minimal TRAb elevation in the early phase after RAI had a higher likelihood of achieving remission.

**Conclusions:**

Decision tree modeling identified early TRAb elevation patterns that serve as strong predictors of RAI efficacy. Incorporating TRAb fluctuations into clinical assessment may facilitate personalized ^131^I dosing strategies, ultimately improving treatment outcomes for GD patients.

## Background

Graves’ disease (GD) is a common autoimmune endocrine disorder and the most frequent cause of primary hyperthyroidism, particularly in women (1). It arises due to the loss of immune tolerance to self-antigens, with the thyroid-stimulating hormone receptor (TSHR) being the main autoantigen (2). Recognition of TSHR peptides by T cell receptors triggers T cell activation, leading to the activation of B cells, which consequently differentiate into plasma cells and produce thyrotropin receptor antibodies (TRAb). These antibodies stimulate thyroid cells, promoting their proliferation and hormone secretion, ultimately resulting in hyperthyroidism (2, 3). In patients with GD, approximately 75%–90% were observed to be TRAb positive (4). Studies have reported varying TRAb level responses after RAI, with some patients experiencing a decrease while others exhibiting an increase (5, 6). The transient increase in TRAb levels in the initial months post-RAI may be attributed to the release of thyroid antigens from damaged thyroid cells. In a small number of patients with onset GD, TRAb levels may remain elevated for years after RAI, suggesting that TRAb production is also related to other triggers, such as age, with some studies suggesting that younger patients may develop higher TRAb levels after RAI (7).

Several factors can impact the effectiveness of RAI therapy, including gender, age, disease duration, iodine uptake, thyroid function, thyroid-related antibodies, and thyroid mass (8–13). However, there remains considerable controversy regarding the specific factors that influence the success of ^131^I therapy for GD. A personalized treatment approach, tailored to these influencing factors, may offer more benefit to patients. To address this, we analyzed the relationship between TRAb levels and treatment outcomes following RAI therapy and developed a decision tree prediction model to forecast RAI efficacy based on these key influencing factors.

## Subjects and methods

### Subjects

A total of 728 patients with GD, treated with ^131^I for the first time at the Tianjin Medical University General Hospital between December 2015 and June 2021, were recruited. Of these, 175 (24%) were male and 553 (76%) were female, with a mean age of 41.9 ± 14.3 years and a median follow-up time of 52 (39, 68) months. Inclusion criteria were as follows: (i) meets the criteria of the ^131^I Graves’ Hyperthyroidism Treatment Guidelines (2013 Edition) (14) and (ii) repeat one or more serum TRAb tests. Exclusion criteria were as follows: (i) patients who had received RAI, (ii) patients who had received radiotherapy to the neck, (iii) patients who had undergone thyroid surgery, (iv) patients with or suspected of having thyroid malignancy, and (v) patients lost to follow-up or with incomplete follow-up data. Free triiodothyronine (FT_3_), free thyroxine (FT_4_), thyroid-stimulating hormone (TSH) levels, thyrotropin receptor antibody (TRAb) levels, highest radioactive iodine uptake (RAIU_max_), effective half-time (T_eff_), and 24-h radioactive iodine uptake (RAIU_24h_) were measured. The study was carried out following the Declaration of Helsinki principles and approved by the Ethics Committee of the General Hospital of Tianjin Medical University (No. IRB2024-YX-532-01). Informed written consent was obtained from all patients.

### Serum assay

Serum circulating FT_3_, FT_4_, and TSH levels were determined by chemiluminescence immunoassay (Abbott Laboratories, Chicago, IL, USA). Serum TRAb was determined by electrochemiluminescence immunoassay (Roche Diagnostics GmbH, Mannheim, Germany). Normal ranges of these parameters were as follows: FT_3_ (2.43–6.01 pmol/L), FT_4_ (9.01–19.05 pmol/L), TSH (0.350–4.940 mIU/L), and TRAb (0–1.75 IU/L).

### Thyroid mass measurement

Thyroid mass was calculated by ultrasound measurements (GE Logiq 400 Pro, GE Healthcare). Measurement of the length (L), width (W), and depth (D) of the left and right thyroid lobes was made using the following formula: thyroid mass (g) = 0.479 × (WR × DR × LR + WL × DL × LL) (15).

### Radioactive iodine therapy and therapeutic grouping

Enrolled patients were treated with RAI after completing relevant laboratory tests. All patients signed a written informed consent for treatment. The therapeutic dose of ^131^I was calculated using the following formula: the dose of ^131^I (mCi) = 0.67 × 100 (Gy/g) × thyroid mass (g)/RAIU_max_ (%) × Teff (d) (16). The predicted absorbed dose to thyroid tissue is 100 Gy/g, and the correction factor is 0.67. Patients received a single oral dose of ^131^I on an empty stomach. Patients were followed up and divided into “Remission” (euthyroid or hypothyroid) and “Remained hyperthyroid” (partial relief, ineffective treatment and relapse) groups according to the guidelines issued by the Chinese Society of Nuclear Medicine (1). The changes in serum TRAb levels (ΔTRAb) were calculated using the following formula: ΔTRAb = (TRAb levels at review − TRAb baseline levels)/TRAb baseline levels.

### Decision tree

In this study, a predictive model was constructed to predict outcomes in patients with rising TRAb after RAI. A decision tree model was constructed to predict the efficacy of the first RAI treatment, based on ΔTRAb 3 months after RAI (ΔTRAb_3_) and ΔTRAb 6 months after RAI (ΔTRAb_6_), respectively (rpart package). Patients with ΔTRAb<0 were excluded from the analysis. Based on the ΔTRAb, this study used the CRAT algorithm to construct a decision tree model with pruning. The algorithm selects the best segmentation points to group the data and uses the Gini index to measure data impurity. The data was randomly divided into a training set (70%) and a test set (30%).

### Statistical analysis

Data were analyzed using SPSS 25.0 statistical software, and the results are presented as the mean ± standard deviation or median (interquartile range). The data were statistically analyzed using two independent samples t-test, Mann-Whitney U-test, and chi-square test. Differences were considered statistically significant when p<0.05. Statistical analyses were carried out with R and the integrated development environment R Studio v 4.3.2 (RStudio, Inc., Boston, MA, USA). For the decision tree analysis, the rpart function of the R package was used.

## Results

### Clinical characteristics of participants

The baseline characteristics of the 728 enrolled patients are summarized in **Table 1**. A total of 326 (44.8%) patients achieved remission after the first RAI treatment. Patients with a shorter disease duration, no prior use of antithyroid drugs (ATD), lower FT_4_ and TRAb levels, reduced thyroid mass, and lower doses of ¹³¹I are more likely to achieve remission. Gender, the presence of comorbid Graves’ ophthalmopathy (GO), age, FT_3_ level, RAIU_max_, and T_eff_ did not significantly affect the efficacy of the first ^131^I treatment in GD patients.

**Table 1:** The clinical characteristics of patients with Remission and Remained hyperthyroid.

Variables	Remission (n=326)	Remained hyperthyroid (n=402)
Gender, male (%)	81 (24.8%)	94 (23.4%)
Age, years	41.8 ± 14.1	42.1 ± 14.2
Course of disease (months)	18 (3, 72)^*^	36 (6, 84)
GO*, present (%)	64 (19.6%)	97 (24.1%)
ATD treatment, yes (%)	228 (69.9%)^**^	327 (81.3%)
Thyroid function		
FT_3_ (pmol/L)	23.8 ± 13.1	23.4 ± 14.1
FT_4_ (pmol/L)	37.9 ± 20.7^*^	42.0 ± 27.7
TSH (mIU/L)	0.004^*^ (0.004, 0.004)	0.004 (0.004, 0.004)
TRAb (IU/L)	16.56 ± 12.51^*^	18.67 ± 14.40
Thyroid uptake		
RAIU 24 h (%)	64.5 ± 10.4	64.1 ± 10.3
RAIU max (%)	73.9 ± 10.7	72.8 ± 10.1
Teff, days	6.0 ± 5.3	5.7 ± 1.6
Dose of ^131^I, mCi	6.2 ± 3.7^**^	7.9 ± 5.0
Thyroid mass, g	32.1 ± 19.2^**^	45.2 ± 31.6

GO*: The patient had Graves’ ophthalmopathy (GO).

ATD, antithyroid medication.

^*^P<0.05.

^**^P<0.001.

### Serum TRAb levels and the first RAI treatment efficacy

**Figure 1:**
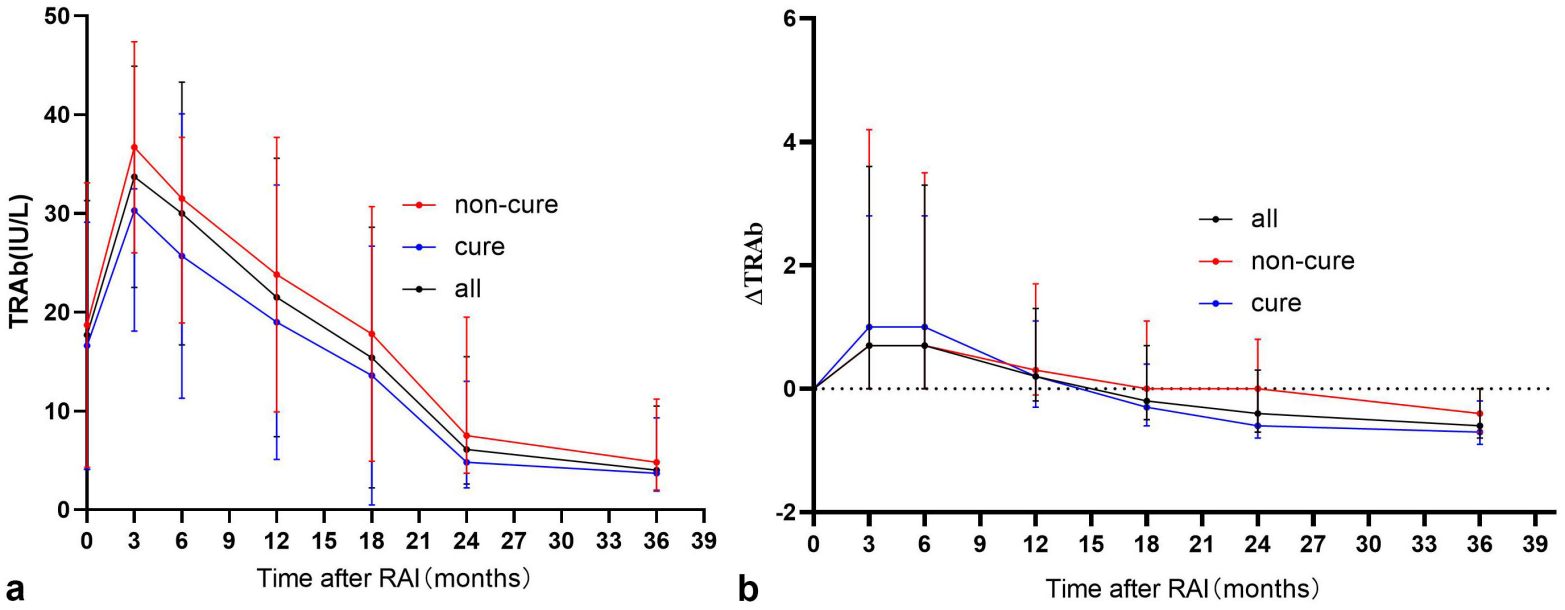
“**(a)** Trends in serum TRAb after RAI treatment” and “**(b)** Trends in serum ΔTRAb after RAI treatment”. The note [ΔTRAb = (TRAb levels at review−TRAb base line levels)/TRAb base line levels].

Prior to RAI, 33 patients were TRAb-negative, of whom 20 (60.6%) eventually achieved clinical cure. TRAb levels showed a significant increase at 3 months post-RAI treatment, followed by a gradual decline (**Figure 1a**). The analysis found that patients with lower serum TRAb levels in 3–18 months after RAI treatment were more likely to achieve remission (**Table 2**). Further analysis revealed that the proportion of patients in the remission group who achieved a reduction in TRAb levels to baseline and normal values after RAI treatment was significantly higher (**Table 3**).

**Table 2:** Serum TRAb levels after RAI in the Remission versus Remained hyperthyroid group.

Time after ^131^I (months)	TRAb of Remission (IU/L)	TRAb of Remained hyperthyroid (IU/L)
3	30.3 ± 12.2^*^	36.7 ± 10.7
6	25.7 ± 14.4^*^	31.5 ± 12.6
12	19.0 ± 13.9^*^	23.8 ± 13.9
18	13.6 ± 13.1^*^	17.8 ± 12.9
24	4.8 (2.2, 13.0)	7.5 (3.7, 19.5)
36	3.7 (1.9, 9.3)	4.8 (2.0, 11.2)

^*^P<0.05.

**Table 3:** Serum ΔTRAb levels after RAI in the Remission versus Remained hyperthyroid group.

Time after ^131^I (months)	ΔTRAb of Remission	ΔTRAb of Remained hyperthyroid
3	1.0 (0, 2.8)	0.7 (0, 4.2)
6	1.0 (0, 2.8)	0.7 (0, 3.5)
12	0.2 (−0.3, 1.1)	0.3 (−0.1, 1.7)
18	−0.3 (−0.6, 0.4)^*^	0 (−0.5, 1.1)
24	−0.6 (−0.8, 0)^*^	0 (−0.7, 0.8)
36	−0.7 (−0.9, −0.2)^*^	−0.4 (−0.7, 0)

ΔTRAb = (TRAb levels at review − TRAb baseline levels)/TRAb baseline levels.

^*^P<0.05.

### ΔTRAb and the first RAI treatment efficacy

**Figure 2:**
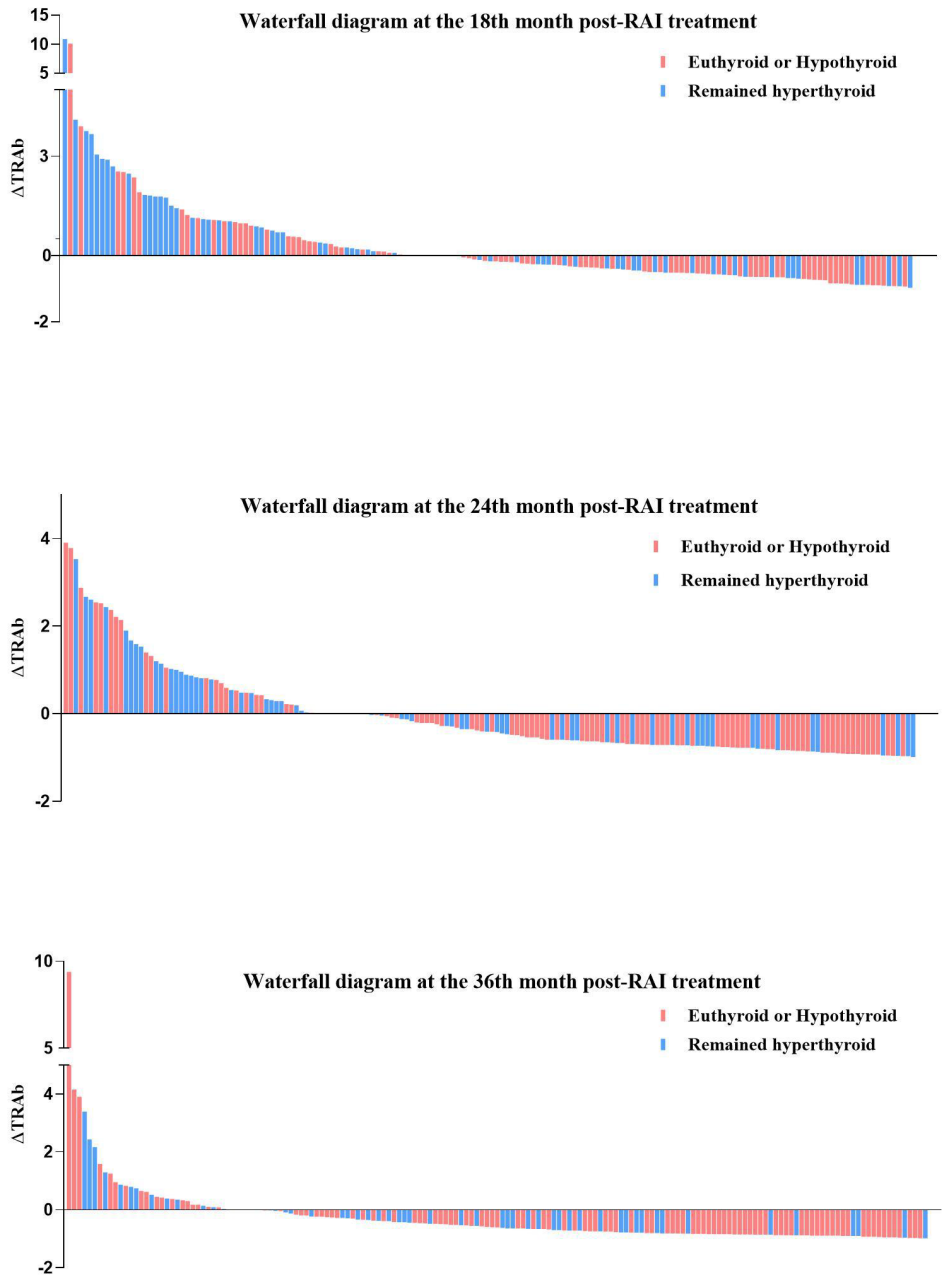
Waterfall diagram at the 18th, 24th, and 36th month post-RAI.

In all enrolled patients, TRAb levels decreased to baseline (ΔTRAb=0) approximately 12–18 months after the first RAI treatment (**Figure 1b**). In the remission and persistent hyperthyroid groups, serum TRAb levels decreased to baseline at approximately 18 months and 24–36 months after the first RAI, respectively (**Figure 1b**). This study showed that patients with a greater decline in TRAb levels over 18–36 months after the first RAI treatment were more likely to achieve remission (**Table 4**). The waterfall plots at 18, 24, and 36 months consistently show a higher proportion of patients in the remission group below the axis (**Figure 2**), indicating that ΔTRAb<0, where serum TRAb was reduced to baseline.

**Table 4:** Whether TRAb levels decreased to baseline values and to normal levels after RAI treatment in the Remission versus Remained hyperthyroid group.

Variable	Whether TRAb levels decreased to normal levels		Whether TRAb levels decreased to baseline values	
	No	Yes	No	Yes
Remained hyperthyroid	371 (92.3%)	31 (7.7%)	264 (65.7%)	138 (34.3%)
Remission	244 (74.6%)	83 (25.4%)	94 (28.7%)	233 (71.3%)
χ^2^	42.681		98.378	
P value	<0.001		<0.001	

### Decision tree

**Figure 3:**
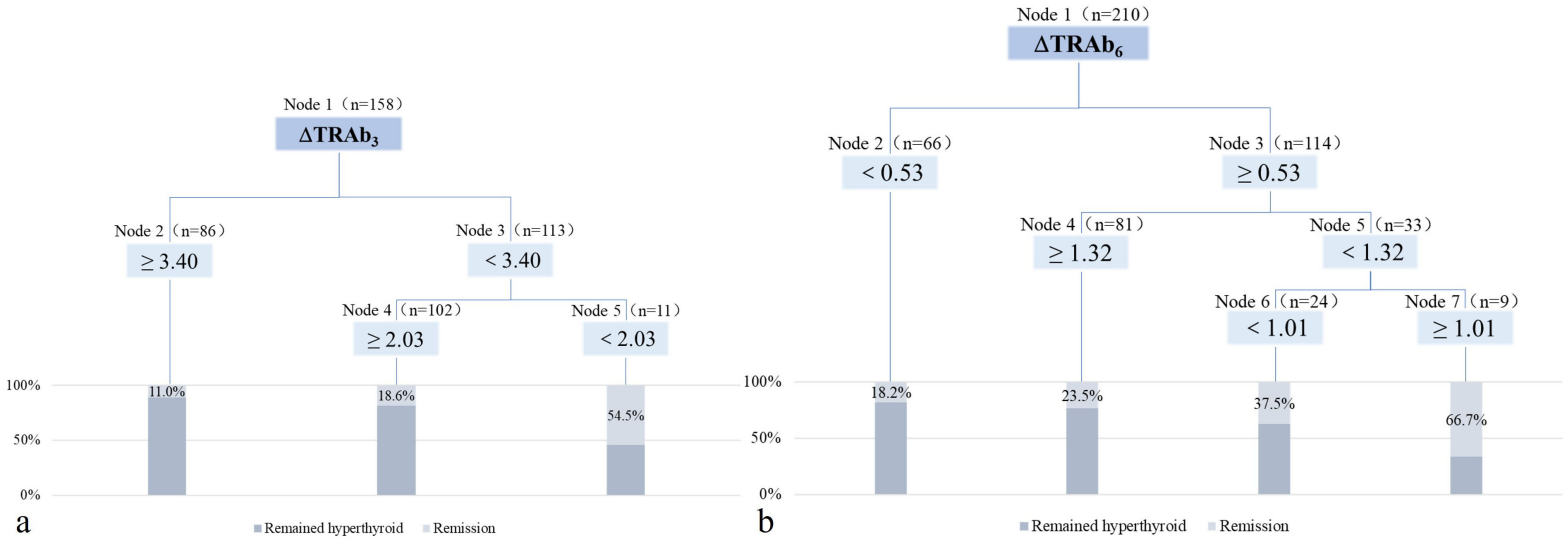
The decision tree model after RAI treatment. **(a)** The decision tree model at the 3rd month post-RAI treatment. **(b)** The decision tree model at the 6th month post-RAI treatment.

The algorithm generated a decision tree with three terminal nodes using ΔTRAb_3_ (**Figure 3a**). According to this decision tree model, patients with ΔTRAb_3_≥3.40 have a remission rate of 11.0%. Patients with 2.03≤ΔTRAb_3_<3.40 have a remission rate of 18.6%, while patients with ΔTRAb_3_<2.03 have a remission rate of 54.5%. The predictive accuracy of this model is 72.46%. The algorithm also generated a decision tree model with four terminal nodes based on ΔTRAb_6_ (**Figure 3b**). According to this model, patients with ΔTRAb_6_<0.53 have a remission rate of 18.2%, while whose with ΔTRAb_6_≥1.32 have a remission rate of 23.5%. Patients with 0.53≤ΔTRAb_6_<1.01 have a remission rate of 37.5%, and those with 1.01≤ΔTRAb_6_<1.32 have a remission rate of 66.7%. The predictive accuracy of this model is 74.36%.

Further analyses were based on decision tree groupings. In the decision tree model at 3 months post-RAI, patients with low ΔTRAb_3_ (cut-off values: 3.40 and 2.03) exhibited high remission rates. These patients were characterized by mild hyperthyroidism, small thyroid mass, and low baseline TRAb levels (**Table 5**). In the decision tree model at 6 months post-RAI, high remission rates were observed in patients with high ΔTRAb_6_ (cut-off value: 0.53). These patients were characterized by mild hyperthyroidism, small thyroid mass, low doses of ^131^I, and low baseline TRAb levels. However, in the subgroup with a cut-off value of 1.32, patients with low ΔTRAb_6_ exhibited higher remission rates. This group was also characterized by mild hyperthyroidism, low doses of ^131^I, and low baseline TRAb levels (**Table 6**). In the subgroup with a cut-off value of 1.01, the smaller sample size resulted in greater variability, preventing further analysis.

**Table 5:** Subgroup analyses according to the decision tree model at the 3rd month post-RAI treatment.

Variable	ΔTRAb_3_		ΔTRAb_3_	
	<3.40	≥3.40	<2.03	≥2.03 and <3.40
Thyroid mass (g)	51.0 ± 35.7^**^	34.0 ± 25.2	52.8 ± 36.5^*^	36.6 ± 24.9
FT_3_ (pmol/L)	25.9 ± 13.6^**^	14.7 ± 8.9	27.1 ± 13.5^**^	16.2 ± 10.2
FT_4_ (pmol/L)	45.9 ± 30.9^**^	31.2 ± 15.8	47.8 ± 31.9^**^	30.9 ± 14.8
^131^I dose (mCi)	9.0 ± 5.5^**^	6.0 ± 4.0	9.3 ± 5.5	6.6 ± 4.6
Age at RAI treatment (years)	39.9 ± 13.3^*^	44.6 ± 14.8	39.7 ± 13.3	41.4 ± 14.8
TRAb (IU/L)	26.4 ± 12.7^**^	4.2 ± 7.3	28.7 ± 11.6^**^	8.2 ± 3.5

* means P<0.05; **means P<0.001.

**Table 6:** Subgroup analyses according to the decision tree model at the 6th month post-RAI treatment.

Variable	ΔTRAb_6_		ΔTRAb_6_	
	<0.53	≥0.53	ΔTR≥1.32	≥0.53 and <1.32
Thyroid mass (g)	56.3 ± 30.1^**^	35.1 ± 20.7	40.3 ± 22.7^*^	33.1 ± 19.6
FT_3_ (pmol/L)	28.9 ± 14.0^**^	18.9 ± 12.0	26.0 ± 13.9^**^	16.3 ± 10.0
FT_4_ (pmol/L)	51.5 ± 32.1^**^	33.7 ± 20.4	42.8 ± 28.3^**^	30.3 ± 15.2
ΔTRAb_3_	0 (0, 0.1)^**^	2.6 (1.2, 6.1)	3.9 (2.5, 8.7)^**^	1.0 (0.8, 1.2)
^131^I dose (mCi)	10.3 ± 5.9^**^	6.1 ± 3.2	7.0 ± 3.3^*^	5.7 ± 3.1
Age at RAI treatment (years)	40.2 ± 14.4	43.1 ± 14.8	43.3 ± 14.5	43.1 ± 15.0
TRAb (IU/L)	33.7 ± 10.0^**^	9.2 ± 6.9	16.9 ± 6.4^**^	6.4 ± 4.4

* means P<0.05; **means P<0.001.

## Discussions

GD is an autoimmune disorder in which autoantibodies target the thyroid TSH receptor, leading to increased thyroid hormone synthesis and secretion (17). Previous study has suggested that TSH binding-inhibition (TBI) could serve as an accurate predictor of the efficacy of RAI therapy (18). However, with advances in the understanding of GD, recent research predicting the therapeutic response to RAI has increasingly focused on various parameters, including age, gender, thyroid volume, thyroid function, the interval between diagnosis and treatment, the administered dose of ¹³¹I, prior antithyroid drug (ATD) therapy and its duration, as well as 24-hour radioactive iodine uptake (RAIU) (8, 19, 20). In this study, TRAb levels were found to be the main factors affecting the efficacy of RAI. Therefore, understanding the factors that influence the efficacy of RAI will help implement personalized treatment plans to minimize the patient’s pain and suffering.

TRAb is closely related to the pathogenesis and diagnosis, including differential diagnosis, of GD. TRAb is a specific polyclonal immunoglobulin produced by thyroid B-lymphocytes. It is classified according to its biological properties into three types: thyroid-stimulatory antibodies (TSAbs), thyroid-blocking antibodies (TBAbs), and neutral TSH receptor antibodies (21). TSAb is most closely associated with GD. It behaves like TSH and stimulates the synthesis of thyroid hormone by binding to the TSH receptor, which leads to hyperthyroidism (22). A study compared three treatment modalities: ATD, thyroidectomy, and RAI. The results demonstrated that ATD and thyroidectomy led to a continuous decrease in TRAb. In contrast, RAI resulted in a significant increase in TRAb, peaking at 3 months, followed by a gradual decline decrease, which in some cases lasted several years (23). Fang et al. (7) found that TRAb levels in most patients undergoing RAI increased during the first year after treatment and then declined. TSAb-positive patients showed a decrease in TRAb levels in the first 1–6 months after RAI, followed by an increase in the next 7–12 months. After 1 year, TSAb levels gradually declined. At one year after RAI, most patients were TBAb positive (7). In this study, we found that TRAb levels increased rapidly within 3 months of RAI treatment, then gradually declined to baseline levels after 12–18 months, which is consistent with previous studies. Moreover, patients with lower serum TRAb levels at 18 months post-RAI and greater TRAb decline between 18–36 months after the first RAI treatment were more likely to achieve remission. The temporary elevation of TRAb shortly after RAI treatment may be due to the transient release of autoantigens from the destruction of thyroid follicular cells. However, the changes observed over a longer period after RAI may be influenced by various factors.

In this study, decision tree models were constructed to predict RAI efficacy based on ΔTRAb_3_ and ΔTRAb_6_, respectively. In the 3-month post-treatment model, despite the presence of multiple subgroups based on ΔTRAb_3_, the overall trend indicated higher remission rates in patients with low ΔTRAb_3_. Further analysis revealed that this group had less severe hyperthyroidism. We hypothesized that these patients experienced less thyroid tissue destruction early after RAI treatment, leading to the production of fewer autoantigens and a smaller increase in TRAb. In the predictive model at 6 months after RAI treatment, patients with ΔTRAb_6_<0.53 showed a low remission rate, with baseline TRAb levels close to 40 U/L (measurement limit). Remission rates were higher in patients with 0.53≤ΔTRAb_6_<1.32, who had mild hyperthyroidism, low baseline TRAb levels, and low ΔTRAb_3_. Therefore, we suggest that patients with milder hyperthyroidism experienced a lower degree of TRAb elevation early after RAI treatment, which was associated with a higher remission rate.

There are some limitations to this study. Firstly, this is a retrospective study, and most patients who could be regularly followed up had some degree of disease persistence (e.g., hyperthyroidism or hypothyroidism). Conversely, those who were cured were gradually lost to follow-up, which led to a low observed remission rate. Secondly, the serum TRAb assay in this study has an upper measurement limit, introducing a degree of error when assessing its increase. Finally, this study only analyzed the effect of serum TRAb on the efficacy of RAI treatment. However, TRAb was further classified into three types, which could provide additional insights not explored in this analysis. Some studies had shown that TSAb stimulates thyroid cell proliferation, leading to increased thyroid hormone synthesis and release, resulting in hyperthyroidism. In contrast, TBAb inhibits thyroid cell function, causing follicular atrophy, reducing thyroid hormone synthesis and release, and leading to hypothyroidism (24). Additionally, it has also been shown that abnormally elevated TRAb levels early after RAI treatment have a limited effect on subsequent disease regression (24). Therefore, the mechanisms underlying these changes should be further analyzed in the subsequent studies on TSAb and TBAb levels after RAI.

Pre- and post-treatment serum TRAb levels, as well as their changes, may influence the efficacy of RAI treatment. Patients with lower serum TRAb levels within 18 months of RAI treatment are more likely to achieve remission. In this study, we constructed two simple yet effective decision tree models to predict RAI efficacy based on the degree of TRAb elevation in the early period following treatment. Both models suggest that patients with high ΔTRAb have a lower remission rate. Further analysis shows that these patients with hyperthyroidism are more serious cases. For these patients, we suggest increasing the dose of ^131^I treatment to enhance therapeutic efficacy and provide better disease relief.

## Data Availability

The raw data supporting the conclusions of this article will be made available by the authors, without undue reservation.
